# A Research of Methamphetamine Induced Psychosis in 1,430 Individuals With Methamphetamine Use Disorder: Clinical Features and Possible Risk Factors

**DOI:** 10.3389/fpsyt.2018.00551

**Published:** 2018-11-06

**Authors:** Hong Gan, Yan Zhao, Haifeng Jiang, Youwei Zhu, Tianzhen Chen, Haoye Tan, Na Zhong, Jiang Du, Min Zhao

**Affiliations:** ^1^Shanghai Mental Health Center, Shanghai Jiao Tong University School of Medicine, Shanghai, China; ^2^Shanghai Key Laboratory of Psychotic Disorders, Shanghai, China

**Keywords:** methamphetamine (MA) use disorder, psychosis, clinical features, risk factors, cognitive function

## Abstract

**Background and Aims:** Methamphetamine (MA) abuse is commonly associated with the development of psychotic symptoms. The predictors and related risk factors of MA induced psychosis (MIP) are poorly understood. We investigated the occurrence of MIP, and analyzed the clinical features and possible risk factors among individuals with MA use disorder

**Method:** One thousand four hundred and thirty participants with MA use disorder were recruited from compulsory rehabilitation centers in Shanghai. A structured questionnaire including demographic characteristics, drug use history, visual analog scales, Beck Depression Inventory-13 (BDI-13), and Hamilton anxiety scale-14 (HAMA-14) were used to collect clinical related information. Fifty-six participants had accomplished the test of CogState Battery.

**Results:** Among the 1430 individuals with MA use disorder, 37.1% were diagnosed as MIP according DSM-IV. There were significant differences in age, marital status, age of drug use onset, MA use years, Average MA use dose, interval of MA use, maximum dose, concurrent use of alcohol, and other drugs, VAS score, MA dependence, BDI-13 scores, HAMA-14 scores, verbal learning memory, and visual learning memory between the MIP group and the none MIP group (*P* < 0.05). The age of drug use onset (OR = 0.978, *p* = 0.011), average drug use dose (OR = 1.800, *p* = 0.015), craving score (OR = 1.069, *p* = 0.031), MA dependence (OR = 2.214, *p* < 0.001), and HAMA scores (OR = 1.028, *p* < 0.001) were associated to MIP.

**Conclusion:** Individuals with MIP had more severe drug use problems, emotional symptoms and cognitive impairment. Earlier onset of drug use, higher quantity of drug use, higher craving, middle or severe drug use disorder and more anxiety symptoms may be related risk factors of MIP.

## Introduction

Drug abuse is a global public health problem, which is supported by 275 million people worldwide (about 5.6 percent of the population during the age of 15–64 years) utilizing drug at least once in 2016, according to the 2017 World Drug Report. And abusers of methamphetamine (MA) have reached 37 million globally, with a significantly increasing use especially in the East and South-East Asia (1). In China, MA has been the most commonly used drug instead of heroin (2).The abuse of MA can cause a series of physiological and mental health problems, including sympathetic excitation, euphoria, energetic, alertness, suspicion, and psychiatric disorder (2).

There is a greater chance that MA causes psychosis symptoms than other addictive substances (3). According to a number of epidemiological studies, it is about 40 percent of MA abusers occurring the psychiatric symptoms (4). Chen et al. (5) compared the individuals with MA use disorder (*n* = 445), among them, there were 174 (39%) participants with a lifetime diagnosis of a MA-induced psychotic disorder; and 261 (59%) without MIP (5). But, Glasner et al. examined 526 individuals who met the DSM—IV criteria for MA dependence, and there were 68 (12.9%) participants with psychotic symptoms and 458 (87.1%) participants without psychotic symptoms at 3-year follow-up (6). And the psychiatric disorder which is caused by MA abusing was called MA induced Psychosis (MIP) (7). Hallucinations and delusions are the main symptoms of MIP, with auditory hallucination and persecutory, reference delusions being the most common symptoms of hallucinations and delusions individually (7). And the delusion is probably associated with mental excitement, increased vigilance, and increased attention after MA use (8).

The psychiatric symptoms in the most patients suffered MIP usually represented to be transient. But there is still a chronic and recurrent course of disease (9). A higher proportion of depression, suicide, antisocial personality, bipolar disorder, cognitive defects, behavioral disorders, and even personality disintegration may appear in MIP participants (10). Gradually, those severe patients are out of touch with society, and finally the social function is completely lost (11). Therefore, it may be considered to be necessary for participants with MIP to obtain the antipsychotic medications (12).

Shalini et al. (13) completed the only comprehensive review to examine correlates of psychosis among people who use MA. They analyzed 20 studies that included 13 populations, and found that there is association among the indices of the quantity of MA use, polydrug use and alcohol dependence in the likelihood of psychotic symptoms. However, the sociodemographic factors, including age, gender and employment status, were not associated with psychosis risk in MA abusers. On the contrary, another study reported that using MA early seemed to be a more valid predictor for psychosis than long-term utilization of amphetamine (5, 14). Moreover, a significant dose-dependent increase in the occurrence of psychotic symptoms during the periods of MA use (15). A recent Norwegian study found no association between the severity of psychotic symptoms and quantitative measures of blood amphetamine concentration (16). In addition, there have been three narrative reviews focusing on risk factors associated with MIP (4, 17, 18), showed that it was complicated to determine causality for the MIP, and difficult to make clear about the risk factors of psychotic disorders among MA users.

Due to relatively small sample size and methodological differences in the existing literature, it is difficult to draw conclusions about the rates or distinguishing features of psychotic disorders among MA users so far. Large sample of clinical studies are needed to understand the clinical features and possible risk factors of MIP.

In view of the increasing abuse of MA, especially MA as the representative drug and the high prevalence of MIP. A research of 1,430 individuals with MA use disorder was conducted, with the aim to understand the prevalence of MIP and its clinical features and possible risk factors.

## Materials and methods

### Participants

MA use disorder participants were recruited from three Compulsory Rehabilitation Centers in Shanghai from October 2012 to June 2014. Eligible participants were required to meet DSM-IV criteria for MA abuse or dependence (MA use disorder) (7); 15–60 years old; men and women not limited; subjects who had organic brain diseases, severe medical conditions, and noncooperation were excluded. And 1,430 MA use disorders, meeting our requirements, were chosen in our research.

The participants with MA use disorder were divided into two groups according to whether they were diagnosed as MIP, and these two groups were defined as the MIP group(*n* = 530) and none MIP group(*n* = 900). All participants were interviewed with a series of scales including the demographic characteristics, drug use history, craving, mood status, and cognitive function.

Written informed consent was obtained from all subjects after a detailed description of the study. All participants or their legal guardians provide written informed consent before they participated in this study. This study protocol was approved by the Institutional Review Board (2011-37R) of Shanghai Mental Health Center.

### Data collection and measurements

Each subject was interviewed and diagnosed by two trained psychiatrists. Eligible participants should meet DSM-IV criteria for MA abuse or dependence (MA use disorder), and clinical verification of the diagnoses was conducted by a senior psychiatrist with more than 5 years clinical practices. And then, participants should complete a series of scales and tests.

1. Demographic characteristics: age, gender, education, ethnicity, marriage, jobs, family history of psychiatric, etc.
2. Drug use history and Craving: MA use status were collected by a drug use history questionnaire, including age of drug use onset, total years of MA use, dose, interval of use MA, etc. Craving was assessed by visual analog scales (VAS), with 0 mm being “no craving” and 100 mm representing “most craving ever experienced for MA.” The subjects were asked to mark the position which represented their craving the most appropriately, and the distance from the 0 mm to the marked position indicated the degrees of craving score.
3. Mood Status: The depression and anxiety status were assessed by Beck Depression Inventory-13 (BDI-13) and Hamilton anxiety scale-14 (HAMA-14), respectively.
4. Cognitive Function: We assessed cognitive function using the Chinese version of the CogState Battery, which is a repeatable and sensitive computerized cognitive test with good validity and reliability (19–21). The eight tasks, including Detection task (DET), Identification task (IDN), International shopping list task (ISLT), One card learning task (OCL), Two back task (TWOB), Social emotional cognition task (SEC), Continuous paired association learning task (CPAL), and The Groton maze learning task (GML), were examined, and the participants needed complete the battery as quickly and accurately as possible (22).

The scores of DET, assessing processing speed, and IDN, assessing attention/vigilance, are the mean of reaction times for correct responses. ISLT, showing the verbal learning and memory, is a 12-word, 3-trial verbal memory test. The score is defined as the total number of correct responses. OCL, TWOB, and SEC represent, respectively the cognitive function of visual learning memory, working memory, and social cognition. Their scores are the proportion of correct responses, denoting the accuracy of performance. CPAL and GML taps, respectively spatial working memory and problem solving/error monitoring. The scores of CPAL and GML tasks are the total number of errors.

These tasks were displayed on a green screen, and standardized instructions provided by trained researchers before each task begins. Results of the CogState Battery were uploaded to a secure account on the CogState server site (http://www.Cogstate.com), on which data were calculated and normalization was transformed.

### Statistical analysis

The data were analyzed by using SPSS, version 19.0. Descriptive statistical analysis were done with the prevalence and clinical characteristics of the participants with MIP. Group differences (MIP group and none MIP group), including the demographic characteristics, drug use history, the mood status and cognitive function, were compared using independent-sample *t*-test or Mann-Whitney U test for continuous variables and chi-square test for categorical variables. The factors of the difference between the two groups were analyzed by pairwise correlation analysis. The multivariate logistic regression analysis were used to explore the possible factors related to the MIP and the OR (95%CI) were be calculated. The statistically significant level were reported with *p* < 0.05 (two-sided tests).

## Results

### The prevalence and clinical features of MIP

A total of 1,430 participants with MA use disorders (697 diagnosed as MA abuse and 721 MA dependence) were investigated, and 530 (37.1%) participants were diagnosed as MIP according to DSM-IV. Among the 530 MIP participants, 403 (76%) individuals were male, 127 (24%) were female. The age was ranged from 17 to 59 years old and the average age was 35.78 ± 9.13 years old.

One hundred and forty nine cases (28.1%) were diagnosed as MA abuse, and 381 cases (71.9%) were diagnosed as MA dependence. In the MIP participants, delusions and hallucinations were the most common symptoms.

**Figure 1:**
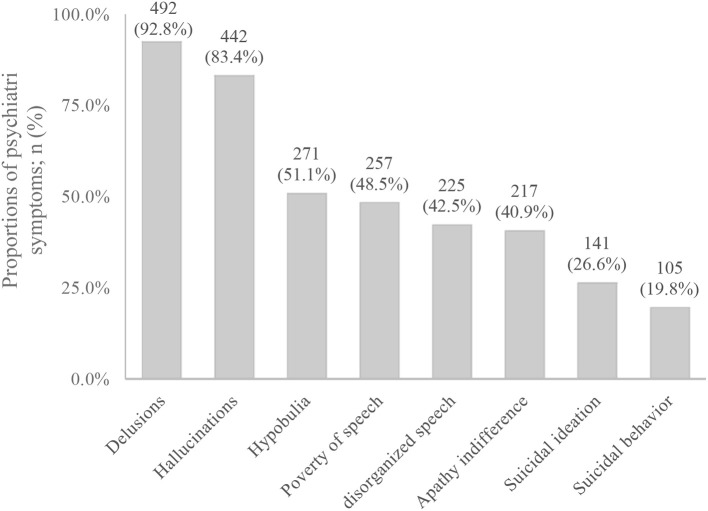
psychiatric symptoms in MIP group.

83.4% had hallucinations, and auditory hallucinations was the most common symptom (79%), 40.6% had visual hallucinations, and 33.6% had tactile hallucination. And 92.8% of MIP participants had delusions, in which delusions of reference was 48.3%, persecutory delusion was 40.6%, grandiose delusion was 23.4%, jealous delusion was 39.5%, and delusions of control was 36.2%. In addition, 51.1% of MIP participants had hypobulia, 48.5% had poverty of speech, 42.5% had disorganized speech, 40.9% had apathy indifference, 26.6% had suicidal ideation, and 19.8% had suicidal behavior (Figure 1). As for the course of the disease, 420 participants had experienced the transient episodes of psychiatric symptoms (with a duration of <1 month), accounting for 81.2%, and 97 participants had experienced the persistent episodes of psychiatric symptoms (with a duration of over 1 month), accounting for 18.8%. However, previous treatment rate was only 7.4%.

### The demographic and drug use characteristics of MIP

The demographic data and drug use history were compared between MIP group and none MIP group. The result showed that there were significant differences in age, marital status, age of drug use onset, MA use years, Average MA use dose, interval of use MA, maximum dose, concurrent use of alcohol and other drugs, VAS score, and MA dependence between the two groups (*P* < 0.05) (Table 1).

**Table 1:** Comparisons of the demographic characteristics and drug use history.

	**MIP group (*n* = 900)**	**None MIP group(*n* = 530)**	**t/z/x^2^**	***P***
**DEMOGRAPHIC CHARACTERISTICS**				
Gender (male)	707 (79.3%)	403 (76.0%)	2.13	0.144
Age (years)	37.65 ± 9.53	35.78 ± 9.13	3.58	<0.001
Edu (years)	7.86 ± 4.08	8.21 ± 3.85	1.60	0.110
Ethnicity (Han)	872 (97.8%)	523 (98.9%)	2.27	0.132
Work status (employed)	492 (54.7%)	285 (53.9%)	0.08	0.772
Marital status (Single or divorced)	367 (41.1%)	274 (52.2%)	16.28	<0.001
Family history of psychiatric disorder	33 (3.9%)	28 (5.4%)	1.58	0.209
**DRUG USE HISTORY**				
Age of drug use onset (years)	32.52 ± 9.71	30.05 ± 9.27	4.67	<0.001
MA use year(years)	5.19 ± 4.02	5.76 ± 3.80	2.59	0.010
Average MA use dose (g)	0.38 ± 0.33	0.49 ± 0.38	6.31	<0.001
Interval of use MA (days)	13.58 ± 13.55	14.57 ± 13.25	2.29	0.022
Maximum dose (g)	0.80 ± 1.33	1.13 ± 1.10	8.50	<0.001
Concurrent use of alcohol	108 (36.2)	69 (48.3%)	5.80	0.016
Concurrent use of other drugs	83 (9.3%)	112 (21.3%)	40.07	<0.001
Route of use (injecting)	181 (21.0%)	125 (24.6%)	1.84	0.175
Craving score	3.14 ± 2.93	3.74 ± 3.03	3.42	0.001
MA dependence	340 (38.3%)	381 (71.9%)	149.91	<0.001

### The BDI, HAMA scores and cognitive functions of MIP

The depression status and the anxiety status were assessed, respectively by BDI-13 and HAMA-14, and the scores of the two scales were significantly different between MIP group and none MIP group (*P* < 0.001). Moreover, in MIP group, the suicidal ideation and behavior were more than none MIP group (Table 2).

**Table 2:** Comparisons of mood status.

	**MIP group (*n* = 900)**	**None MIP group (*n* = 530)**	**t/x^2^**	***P***
HAMA scores	13.13 ± 12.51	20.28 ± 13.74	8.09	<0.001
DBI scores	9.76 ± 7.71	13.21 ± 8.51	6.27	<0.001
Suicidal ideation	68 (7.7%)	141 (26.6%)	94.81	<0.001
Suicidal behavior	41 (4.6%)	105 (19.8%)	82.96	<0.001

Fifty six participants had accomplished the test of CogState Battery, including 24 MIP cases and 30 none MIP cases. Independent-sample test comparing the performance between MIP group and none MIP group were conducted on the eight cognitive tests. The significant differences were found on the tasks of ISL (*t* = 2.62, *p* = 0.011) and OCL (*t* = 2.34, *p* = 0.023) between the two groups (Table 3).

**Table 3:** Comparisons of cognitive function.

	**MIP group(*n* = 24) (Mean ± std)**	**None MIP group(*n* = 30) (Mean ± std)**	**t/x^2^**	***P***
DET	2.54 ± 0.12	2.55 ± 1.56	0.28	0.781
IDN	2.71 ± 0.63	2.71 ± 0.89	0.20	0.840
ISL	17.29 ± 4.98	20.77 ± 4.72	2.62	0.011
OCL	0.96 ± 0.89	1.02 ± 0.11	2.34	0.023
TWOB	1.19 ± 0.18	1.19 ± 0.19	0.02	0.988
SEC	1.04 ± 0.20	1.07 ± 0.21	0.67	0.506
GML	68.21 ± 31.14	70.80 ± 23.54	0.35	0.729
CPAL	113.21 ± 60.12	112.00 ± 50.14	0.08	0.939

### The possible risk factors of MIP

The significant different factors between the two groups were analyzed by pairwise correlation analysis, the results found that most variables have the correlation. We selected the independent variable included the age of drug use onset (0 = less than the average of 36 years, 1 = more or equal to 36 years), MA use years (0 = less than the average of 6 years, 1 = more or equal to 6 years), Average MA use dose(0 = less than the average of 0.49 g, 1 = more or equal to 0.49 g), craving score (0 = less than the average of 3.74 scores, 1 = more or equal to 3.74 scores), MA dependence (0 = MA abuse, 1 = MA dependence), HAMA scores (0 = less than the average of 21 scores, 1 = more or equal to 21 scores), and DBI scores (0 = less than the average of 14 scores, 1 = more or equal to 14 scores). And a multivariate logistics regression analysis was carried, and the results showed that the age of drug use onset, Average MA use dose, craving score, MA dependence, and HAMA scores were the risk factors to MIP (*P* < 0.05) (Table 4).

**Table 4:** The multivariate logistic regression analysis of the possible risk factors related to the MIP.

	**B**	**S.E**.	**Wals**	**df**	***P* value**	**OR**	**95%CI**	
Age of drug use onset	−0.022	0.009	6.447	1	0.011	0.978	0.961	0.995
MA use years	−0.026	0.022	1.353	1	0.245	0.974	0.933	1.018
Average MA use dose	0.588	0.241	5.940	1	0.015	1.800	1.122	2.889
Craving score	0.067	0.031	4.641	1	0.031	1.069	1.006	1.137
MA dependence	0.795	0.182	19.139	1	<0.001	2.214	1.551	3.161
HAMA scores	0.028	0.008	13.422	1	<0.001	1.028	1.013	1.044
DBI scores	0.017	0.012	1.897	1	0.168	1.017	0.993	1.041

## Discussion

In the study, we found the a high prevalence of psychiatric symptoms in MA use disorder patients, and the participants with MIP were more severe drug use problems, emotional symptoms and cognitive impairment. Earlier onset of drug use, higher quantity of drug use, higher craving, middle or severe drug use disorder and more anxiety symptoms were related with MIP.

We found that the incidents of MIP was 37.1% in the sample. This was similar to about 40 percent of reported psychiatric symptoms in MA abusers (4). While the prevalence had clearly discernible regional differences. A cross-country study in Australia, Japan, Philippines and Thailand had reported much higher rates of MIP in MA abusers (77.4%), and another higher risk report were from Malaysia (47.95%) (23). In contrast, there were lower risk reports in the U.S 26.5% (24), Sweden (31.5%) (25). A possible explanation was that MA were more popular and purer in the Asia-Pacific region (23).

Among them, the vast majority of MIP participants had hallucinations (83.4%) and delusions (92.8%). The result was similar to the previous studies, which also showed the psychiatric symptoms were mainly hallucinations and delusions (23). Seeing from the studies of recent years, it is complex and multiple to determine the mechanisms of MA causing psychotic symptoms. Studies have shown that MA can quickly across the blood-brain barrier after getting into the blood circulation. Then, it could increase the dopaminergic neurotransmitter in the mesencephalon-cortex pathway, and the glutamatergic neurotransmitters from the cortex to substantia nigra striatum and mesencephalic limbic system, while excessive dopamine and glutamate in the cerebral cortex exceeds the inhibition of γ-aminobutyric acid, which makes psychotic symptoms appear (26–30).

However, previous treatment rate was only 7.4% in our study, being similar to a recent survey. The number of people treated was <10 percent of the estimated drug abusers in China's Yunnan province (31). And the treatment rate was also not optimistic in the U.S, it is reported only 10.6% of substance abusers who needed to be treatment had been treated in 2016 (32). Some of the possible barriers to access to treatment may include the social stigma of drug users, the inconvenience and cost of receiving treatment, and the fear of imprisonment for using illegal drugs (31).

A newer population-based study found that recreational use of MA increases the risk of psychotic symptoms by two to three times (15, 33). However, the probable risk factors for psychotic symptoms are still not fully understood. In this study, we observed that there are different in drug use problems, emotional symptoms, and cognitive impairment between the MA abuser with psychosis and without psychosis. Earlier onset of drug use, higher quantity of drug use, higher craving, middle or severe drug use disorder and more anxiety symptoms were the risk factors to MIP. The results have been supported by some earlier studies. When measuring levels of MA exposure, early onset MA use seemed to predict mental illness more effectively than long-term use (5, 14). It was reported that there was a significant dose-dependent increase in the occurrence of psychotic symptoms during MA use (15). While, according to the World Drug Report (1), utilization of drug by teens is rising, being a serious social problem for adolescent to take drugs with following characters: increasing numbers, gang taking, low cultural level, and so on. Therefore, it is particularly important to carry out anti-drug education and related social work for teenagers and parents. Higher quantity of drug use represents more occurrence rate in MA abusers. McKetin et al. found that the likelihood of experiencing psychotic symptoms was 5 times higher during periods of MA use than during periods of no use, with evidence of a strong dose-response effect (15). A recent systematic review found the similar risk factors (13). One possible reason for the dose-related psychotic symptoms was the enhanced dopaminergic transmissions due to MA (34). Higher craving and middle or severe drug use disorder showed the patients with MA were more dependent on MA. Our results were similar to a systematic review (13) which combined three concept areas: MA or amphetamine, psychosis and risk factors showed that the most consistent correlates of psychotic symptoms were dependence on MA. There was an association between increasing severity of MA dependence and MAP symptoms across four studies (35–37). In comparison to non-dependent individuals, MA-dependent individuals were estimated to have between 2 and 3 times greater odds of developing MAP (35, 38).

In addition, most MIP participants were diagnosed as MA dependence in this study. An early Australian study (39) showed that MA dependent were more three times likely to develop psychotic symptoms than non-dependent counterparts, even after adjusting for schizophrenia and other psychotic disorder history. In the MA dependent participants, as the tolerance increases and withdrawal Symptoms, they require even larger doses of the drug, which has higher risk to lead to psychotic symptoms (15). Another possible explanation is that the dependent MA users' were sensitive to the MA drug, and the psychotic symptoms are more likely to catch them (40).

In our study, more participants with MIP comorbid anxiety and depression. In addition, the MIP participants had higher occurrence rate of suicidal ideation and behavior. The similar results had been reported (36, 41). MA dependence is characterized by affective impairment (42), irritability and emotional reactivity, reduced inhibition (41, 43). Moreover, the anxiety disorder was a risk factor for MIP was also found. The findings concurred with the results found in a study by Chou et al. (44). However, the reasons for this association are not clear. Here are probable reasons as follows: (a) drug abuse is a symptom of dysthymia; (b) drug abuse is an attempt to self-treat symptoms of dysthymia; (c) drug abuse leads to dysthymia; and (d) drug use and dysthymia share a common risk factor (45). In fMRI studies, Anne Uhlmann (46) found that MAP patients showed thinner cortices in the fusiform and inferior temporal gyrus (ITG), orbitofrontal (OFC) and inferior frontal gyrus (IFG), and insula, which involved in emotional regulation, compared to the MA group. Therefore, one possible interpretation of our result is that the nerve damage of emotional regulation gyrus is more serious in the participants with MAP.

We observed significant differences on the tasks of ISL and OCL, which represent the verbal and visual learning memory, respectively, between MIP group and none MIP group. The result showed that the participants with MIP perform worse in the verbal and visual learning memory. The Cognitive impairment is a main symptom dimension of MA participants. Current research indicates that long-term use of MA is impaired in learning, memory, executive function, response inhibition, social emotional cognition, and many other cognitive functions (10, 47). Chronic MIP participants had been associated with moderate deficits in learning and memory, which is attributed to abnormal dopamine energy in frontal striatum (10, 48, 49). And abnormal dopamine energy has been probed to be linked with psychosis (28). But the sample size of the participants who accomplished the tests of cognitive functions was small. Therefore, some negative results could be caused by type II error. More number of large sample studies on the cognitive function should be needed in the future.

Our study also has several limitations. First, because the MA use history and the psychosis symptoms have been collected by the self-report, there may be a certain memory error in this retrospective study. Second, some participants had used other substances at least once in this study, and it may be a risk factor of psychosis. But those participants did not meet the standard of substance abuse. MA was the major abuse substance in our participants, and was higher psychosis risk than the other psychoactive substance (33, 37, 39). Even so, the participants with only MA abuse will be needed in the future. Moreover, although the possible risk factors of the MIP have been discussed, but this study was a cross-section study and there was no clear evidence to prove the causal relationship. At the same time, the specific sample sources (the compulsory rehabilitation centers) make this result have certain limitations on the representative population. Therefore, multi-center follow-up cohort studies may help us better explore the risk factors of MIP. Third, Hellem found that mood status may be related to the duration of withdrawal (50), but our data did not contain the duration of withdrawal. Because the participants with MA abuse were interviewed within one month of admission (the last time of substances abuse). Therefore, the participants had similar duration of withdrawal. In addition, this study would not explore the relationship between the mood status and duration of withdrawal. Nevertheless, we should supplement the duration of withdrawal data in the future.

## Conclusion

This study found that there was a high prevalence of psychiatric symptoms in MA use disorder patients, and these symptoms were mainly hallucinations and delusions. Among those participants who were diagnosed as MIP, there were more severe drug use problems, emotional symptoms and cognitive impairment. At the same time, we also found the associated risk factors of MIP were earlier onset of drug use, higher quantity of drug use, higher craving, middle or severe drug use disorder and more anxiety symptoms. These results can help us better understanding the MIP and make the treatment and prevention more targeted. However, in the future, the multi-center follow-up cohort studies should be conducted to explore the clear causal relationship.

## Data accessibility

The datasets generated and analyzed during the current study are available from the first author e-mail: 710931688@qq.com

## Ethics statement

The study was approved by the Ethics Committee of Shanghai Mental Health Centre (Approval number given by the Ethics Committee: 2011-37R).

## Author contributions

MZ, HJ, and JD participated in the study design process, and revisions of the drafts and the final paper. HG, YaZ, YoZ, TC, HT, and NZ recruited subjects and evaluated the clinical symptoms and cognitive function. HG and YaZ analyzed the data and wrote the draft. All authors read and agreed upon the final version of this article.

### Conflict of interest statement

The authors declare that the research was conducted in the absence of any commercial or financial relationships that could be construed as a potential conflict of interest. The reviewer SL and handling editor declared their shared affiliation at the time of the review.
